# Editorial: Utility of protein aggregation assays from laboratory to clinical application

**DOI:** 10.3389/fnagi.2022.998136

**Published:** 2022-08-16

**Authors:** Alison Jane Ellen Green

**Affiliations:** The National CJD Research and Surveillance Unit, Centre for Clinical Brain Sciences, University of Edinburgh, Edinburgh, United Kingdom

**Keywords:** RT-QuIC, PMCA, seed amplification assays, prion disease, α-synuclein

The development of protein amplification assays, more accurately known as seed amplification assays (SAA), has been one of the most significant advances in the diagnosis and understanding of neurodegenerative diseases associated with the deposition of misfolded proteins. The most widely used SAAs are real-time quaking induced conversion (RT-QuIC) and protein misfolding cyclic amplification (PMCA). These assays were initially used to investigate misfolded prion protein (PrP) in prion disorders, but their application has been extended to additional proteins associated with other proteinopathies such as α-synuclein (α-syn), beta-amyloid (Salvadores et al., 2014), tau protein and TAR DNA-binding protein 43 (TDP-43). Both RT-QuIC and PMCA are able to amplify minute amounts of disease-associated proteopathic seeds in accessible biofluids and tissues such as cerebrospinal fluid (CSF), olfactory mucosa (OM) and skin with a high degree of sensitivity and specificity. The high sensitivity and specificity of CSF RT-QuIC PrP in sporadic Creutzfeldt-Jakob disease (sCJD), the commonest form of human prion disease, has led the European Center for Disease Control (ECDC) and the US Centers for Disease Control and Prevention (CDC) to introduce RT-QuIC analysis of CSF or other tissues as a criterion for diagnosis of probable sCJD.

This Research Topic aims to bring together clinical and basic scientists to review the latest developments in the utility of SAA in terms of diagnosis, disease stratification and assessing zoonotic potential of emerging animal transmissible spongiform encephalopathies (TSEs).

CSF RT-QuIC has a sensitivity of 79–100% for the diagnosis of sCJD (Poleggi et al.; Vascellari et al.; Coysh and Mead), with larger studies reporting a sensitivity of over 90%. CSF RT-QuIC is less sensitive in rare sub-types of sCJD, variably protease sensitive prionopathy and in uncommon forms of genetic CJD and prion disease. A smaller number of studies have utilized skin and OM as alternatives to CSF and have demonstrated comparable sensitivity to CSF. Larger studies will be needed to confirm these findings. There is a wide range of methodologies in use, with different recombinant substrates being used. Despite these variations, several multi-center studies have shown excellent agreement between laboratories.

The success of prion RT-QuIC assays prompted the development of SAAs for other proteinopathies. The development of SAAs for α-synucleinopathies has been particularly successful. Studies using CSF reported sensitivities and specificities of 84–95% and 82–100% for Parkinson's disease (PD) and 92–195% and 94–100% for Lewy body dementia (LBD; Vascellari et al.; Nakagaki et al.). In contrast, the sensitivity of these assays in Multi-System Atrophy (MSA) is more variable (35–86%) and this has been attributed to differences in the conformation of misfolded α-syn in MSA when compared to PD or LDB. Interestingly, CSF α-syn RT-QuIC is positive in 90% of patients with idiopathic rapid eye movement (REM) sleep behavior disorder (iRBD). This is important as patients with iRBD often progress to either PD or LBD. Interestingly the detection of a positive CSF α-syn RT-QuIC preceded the development of PD or LBD with a mean of 3.2 years2 (Iranzo et al., 2021), suggesting the CSF RT-QuIC may be used as an early marker of disease.

The detection of aggregated α-syn by RT-QuIC in skin tissue from PD patients has comparable sensitivity and specificity to that of CSF (Nakagaki et al.). However, the sensitivity may vary depending on the location of the skin biopsy, with cervical skin biopsy samples showing a higher sensitivity than leg or abdominal skin biopsies. Although, the use of OM as a potentially diagnostic tissue has shown a considerable diagnostic accuracy in patients with DLB it has been less promising in PD.

The development of SAAs offers an opportunity for a significant improvement in ante-mortem preclinical diagnosis of these neurodegenerative disorders. Not only is this a major advance for clinical care, it also has the potential to improve recruitment into clinical trials. Clinical trials are hindered by poor accuracy of ante mortem diagnosis, lack of detection of co-pathologies and late recruitment into trials. The use of SAAs can identify the relevant proteopathic seeds underlying the pathology, enabling clinical trials into therapeutic agents targeting the pathobiology of the underlying neurodegenerative diseases more effective (Coysh and Mead). Using a panel of SAAs underlying co-pathologies can also be identified which will aid the stratification of patients within clinical trials.

In addition to clinical diagnosis, SAAs are important tools for research into neurodegenerative diseases. In particular, the use of PMCA in prion disease has been extremely valuable. Peden et al. describe the use of serial PMCA to assess the zoonotic potential of animal prion diseases as an effective and quicker alternative to animal transmission studies. Cazzaniga et al. reports the use of PMCA analysis of OM in sCJD patients to identify the molecular type of the abnormal PrP found in these patients. Previously, this information was obtained by Western Blot analysis on brain tissue of patients deceased for CJD or from a brain biopsy.

In summary, the articles included in this Research Topic highlight the growing importance and value of SAAs in both research and clinical diagnosis of the many neurodegenerative diseases associated with underlying proteinopathy. Future application of SAA will consist in determining for each patient a proteinopathy profile and to address personalized therapies.

## Author contributions

The author confirms being the sole contributor of this work and has approved it for publication.

## Funding

This work was funded by Department of Health and Social Care Policy Research Programme and the Scottish Government (PR-ST-0614-00008).

## Conflict of interest

The author declares that the research was conducted in the absence of any commercial or financial relationships that could be construed as a potential conflict of interest.

## Publisher's note

All claims expressed in this article are solely those of the authors and do not necessarily represent those of their affiliated organizations, or those of the publisher, the editors and the reviewers. Any product that may be evaluated in this article, or claim that may be made by its manufacturer, is not guaranteed or endorsed by the publisher.
